# Supplementary material for: Determining the Impact of Heatwaves on Emergency Ambulance Calls in Queensland: A Retrospective Population-Based Study
Source: Int J Environ Res Public Health. 2023 Mar 10;20(6):4875. doi: 10.3390/ijerph20064875 (PMC10049657; doi:10.3390/ijerph20064875)
Supplement: Supplementary file 1 [file ijerph-20-04875-s001.zip › ijerph-2220669-supplementary.pdf]

Figure S1- Number of heatwave days per postcode (Queensland; 2010-2019).

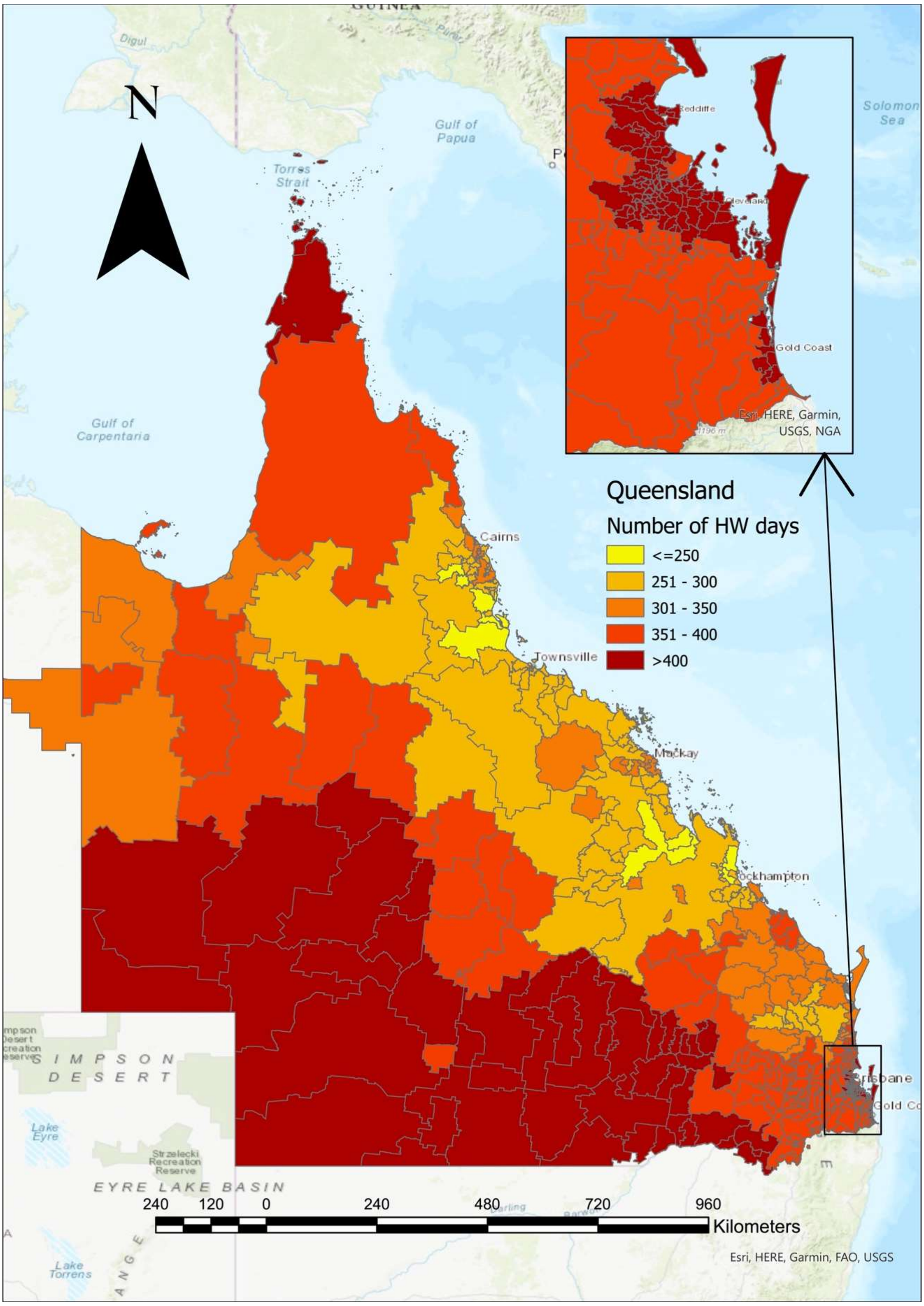

Supplementary Table 1- Suggested broad MPDS categories

Cardiac	9 - Cardiac or Respiratory Arrest / Death<br>10 - Chest Pain / Chest Discomfort (non-traumatic);<br>19 - Heart Problems (AICD)<br><br>[note: there is some crossover between subgroups of card 9 and other categories (e.g., intentional trauma resulting in cardiac arrest (which could be coded to mental health or injury), or unintentional trauma resulting in cardiac arrest (which could be coded to injury).
Stroke	28 -Stroke (CVA) / Transient Ischaemic Attack (TIA)
Mental Health	25 -Psychiatric / Abnormal Behaviour / Suicide Attempt.
Specified Medical	1 - Abdominal pain/problems;<br>2 – allergies (include only 02E01, 02D01, 02D02, 02C01, 02C02, 02B01, 02A01, as other codes relate to envenomation);<br>5 - back pain (non-traumatic or non-recent trauma);<br>12 - convulsions/fittings<br>13 - diabetic problems;<br>16 – eye problems (only 16D01, 16A03)<br>18 - headache;<br>21 haemorrhage (medical related) – includes only 21C01, 21C02, 21C03, 21B03, 21B04, and any codes with suffix M (others are trauma related)<br>26 - sick persons (specific diagnosis)<br>31 - unconscious fainting (near);<br>32 - unknown problems (collapse, 3rd party);
Obstetric	24 – pregnancy / childbirth/ miscarriage
Respiratory	6 - breathing problems;

Injuries	2 – allergies/bites/stings (envenomation) – this includes only 02D03, 02D04, 02D05, 02D06, 02D07, 02A02 [all other 02 codes are most likely allergies]<br>3 – animal bites/attacks;<br>4 – assault/sexual assault;<br>7 – burns (scalds) / explosion (blast);<br>8 – hazardous exposure (carbon monoxide, inhalation / chemical, biological, radiological, nuclear);<br>11 – choking;<br>14 – drowning/near drowning /diving/scuba incident;<br>15 – electrocution / lightning;<br>16 – eye injuries [includes 16B01, 16A01, 16A02); [other 16 codes are not injuries]<br>17 – falls;<br>21 – haemorrhage/lacerations; [this **excludes** 21C01, 21C02, 21C03, 21B03, 21B04, and any codes with suffix M, as these are all medically related, not injury-related]<br>22 – inaccessible incident /other entrapments (non-traffic)<br>23 – overdose/poisoning (ingestion);<br>27 – stab / gunshot / penetrating trauma;<br>29 – traffic/transport incidents<br>30 – traumatic injuries (specific)
Heat/cold exposure	Codes with suffix H are heat-related; codes with suffix C are cold-related

Figure S2- Number of heatwave days per postcode (Queensland; 2010-2011).

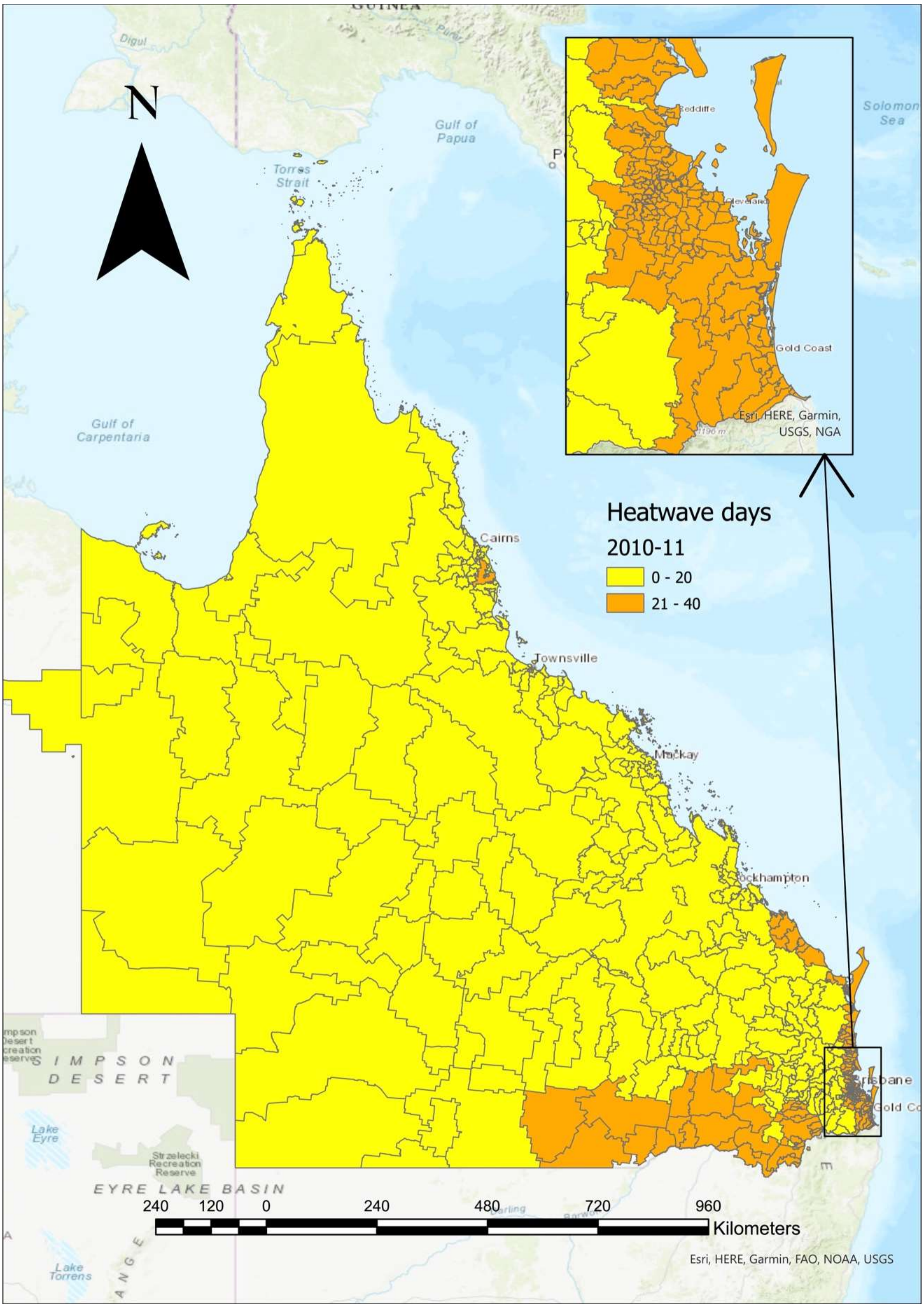

Figure S3- Number of heatwave days per postcode (Queensland; 2011-2012).

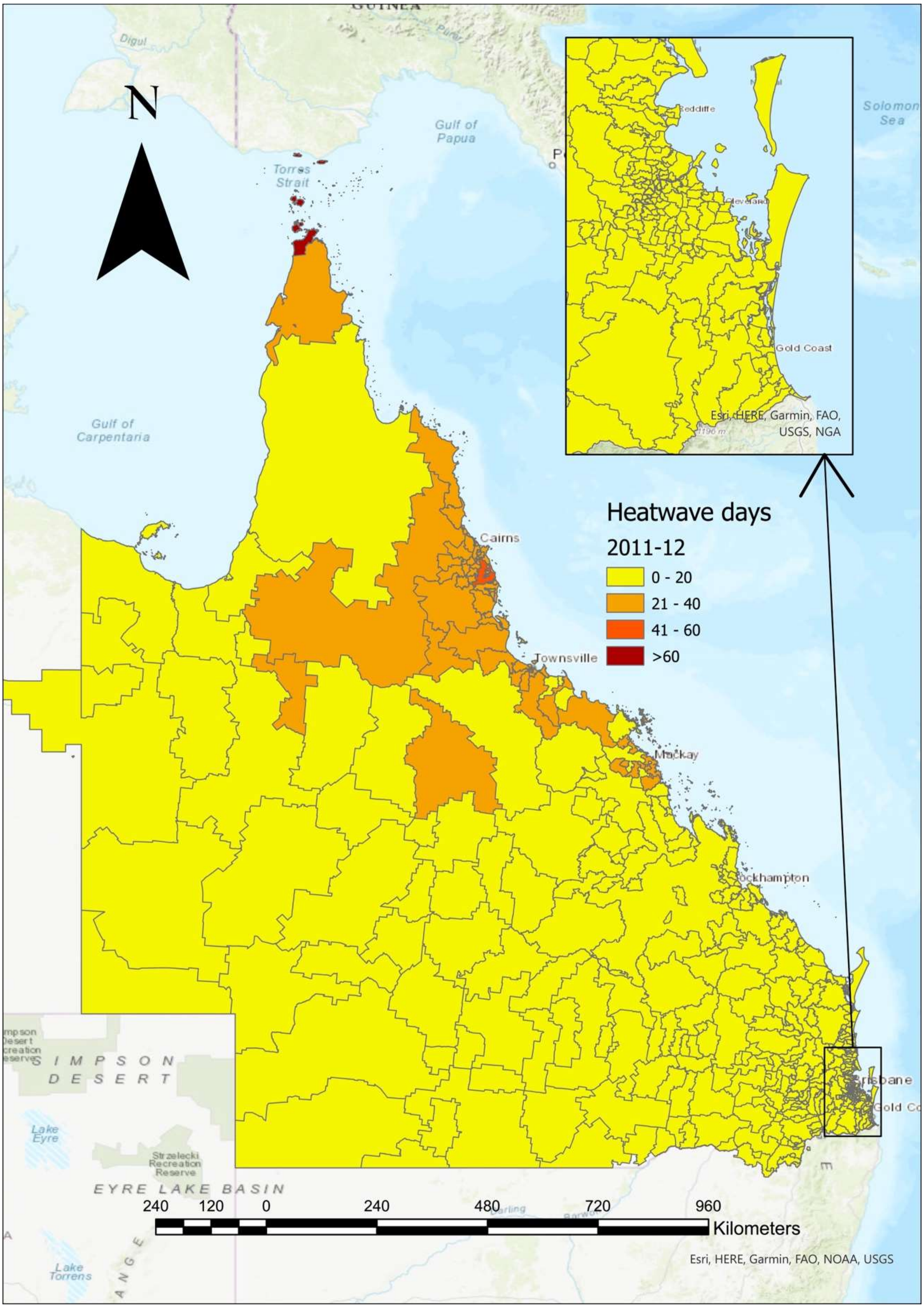

Figure S4- Number of heatwave days per postcode (Queensland; 2012-2013).

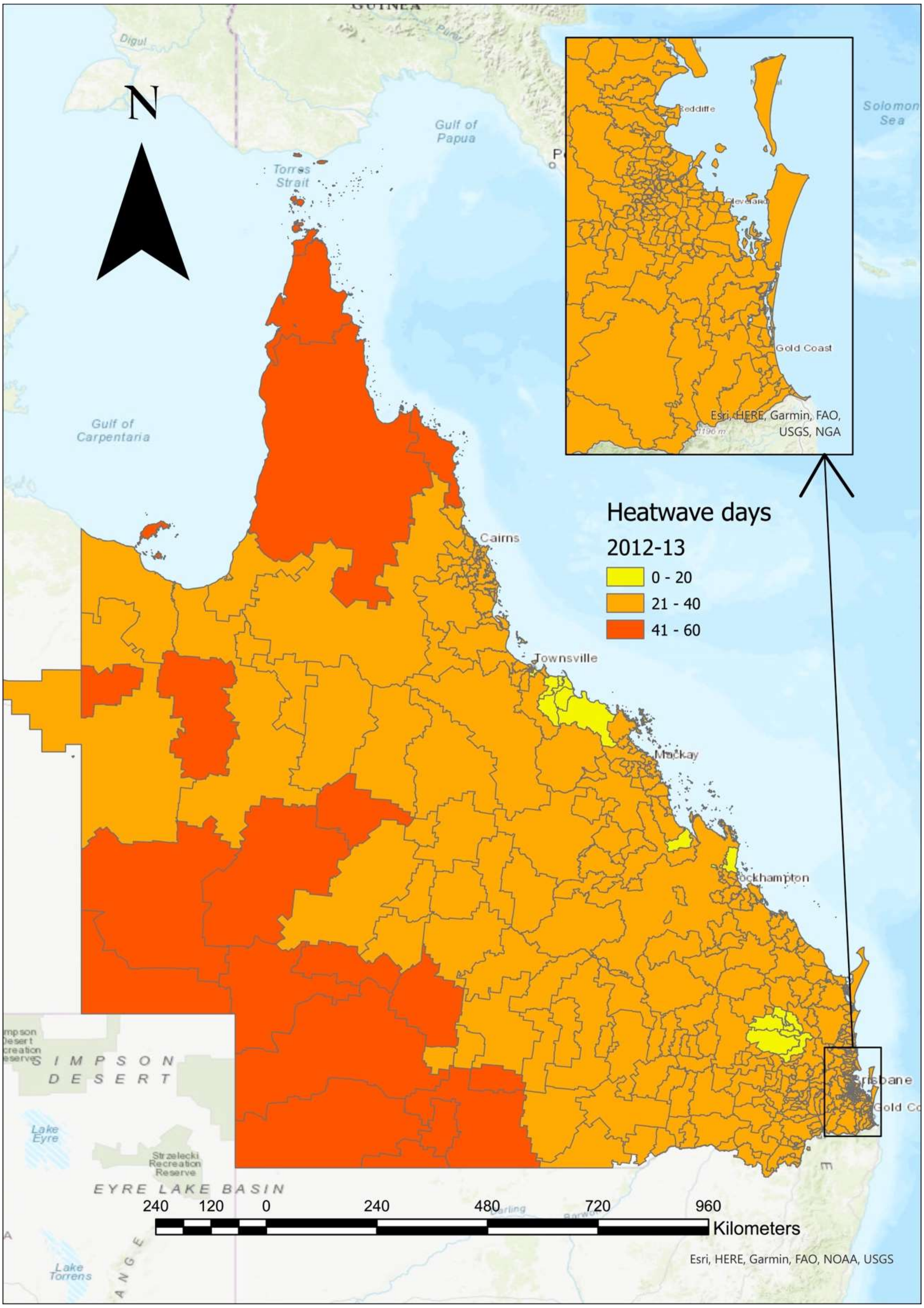

Figure S5- Number of heatwave days per postcode (Queensland; 2013-2014).

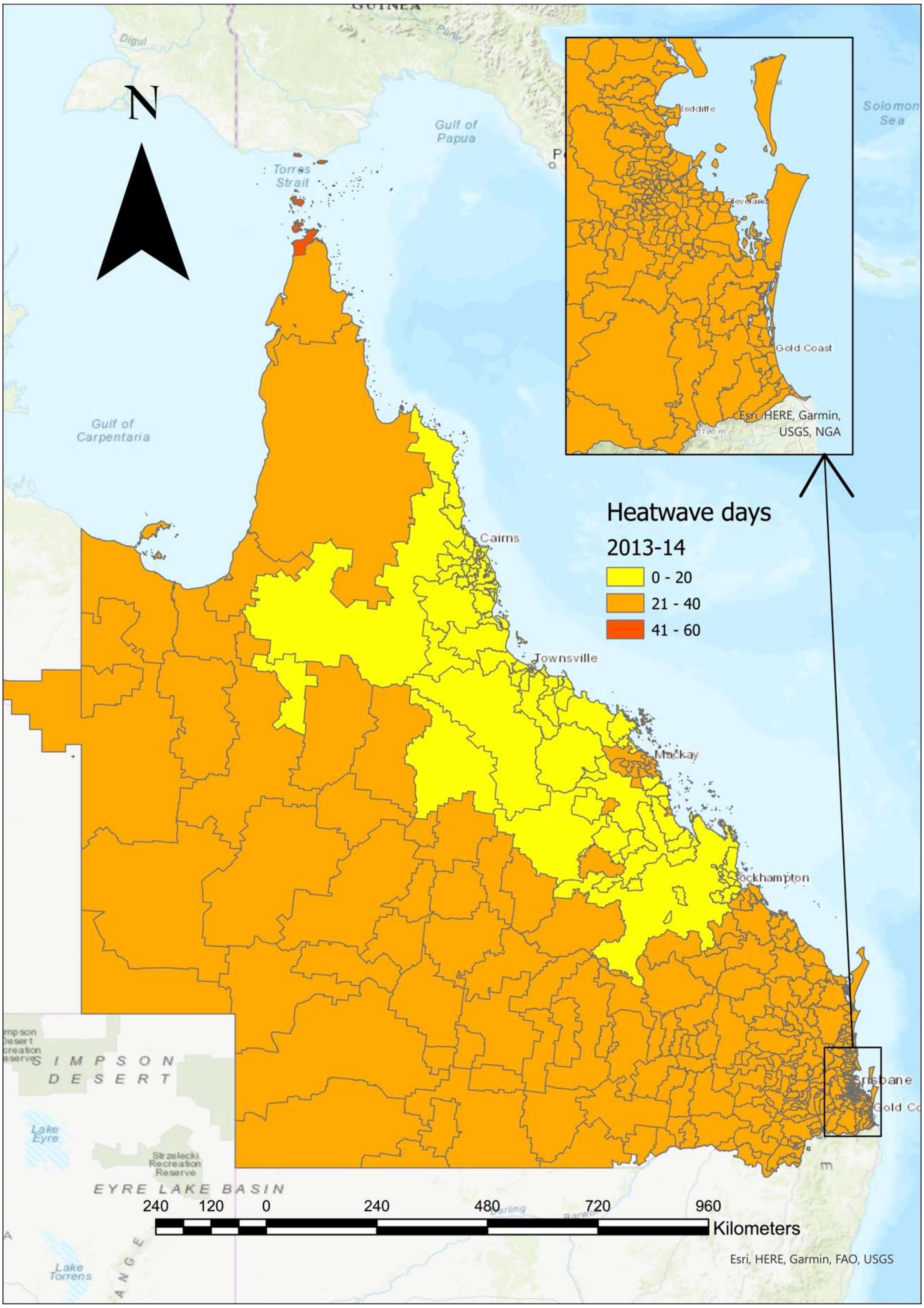

Figure S6- Number of heatwave days per postcode (Queensland; 2014-2015).

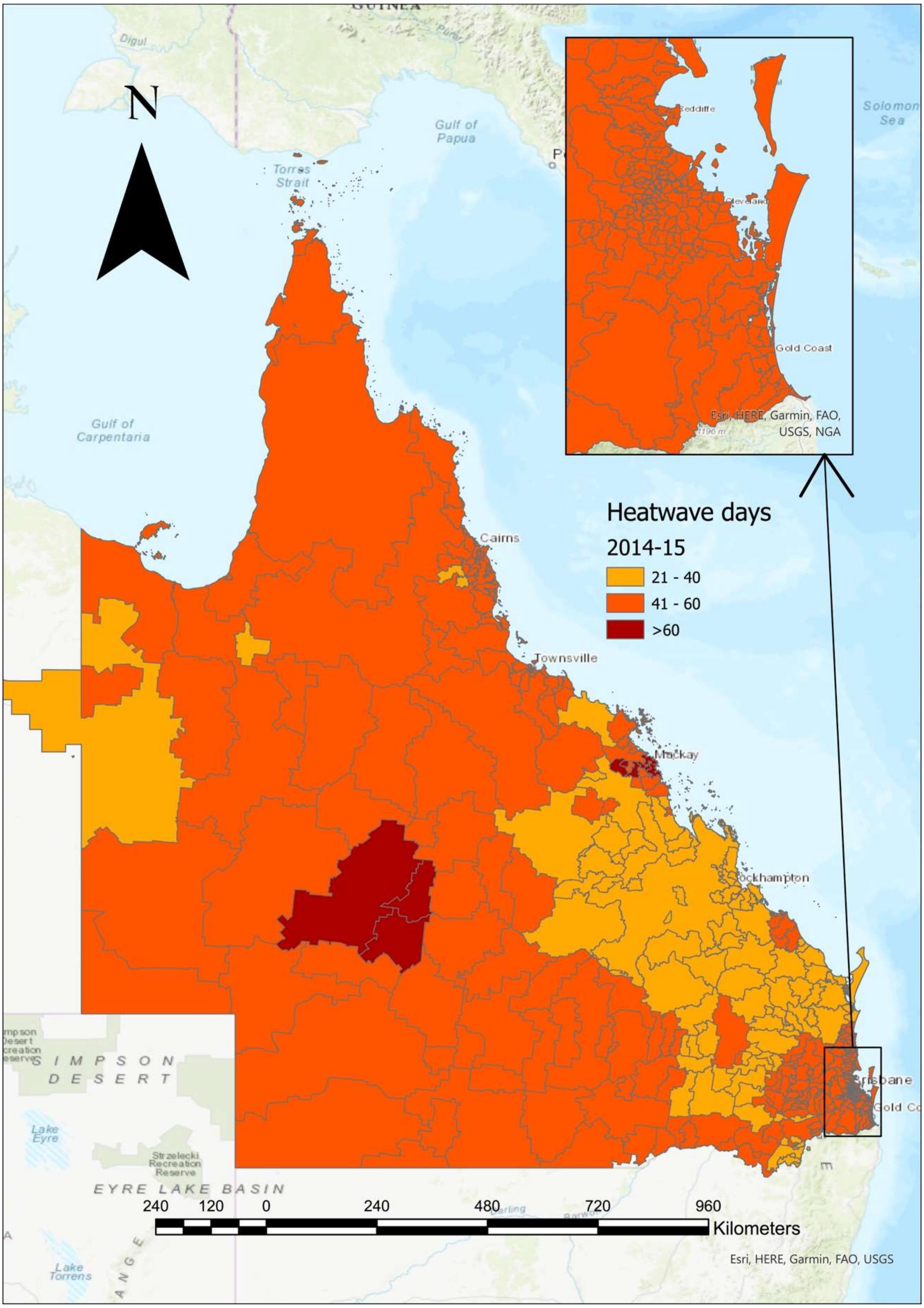

Figure S7- Number of heatwave days per postcode (Queensland; 2015-2016).

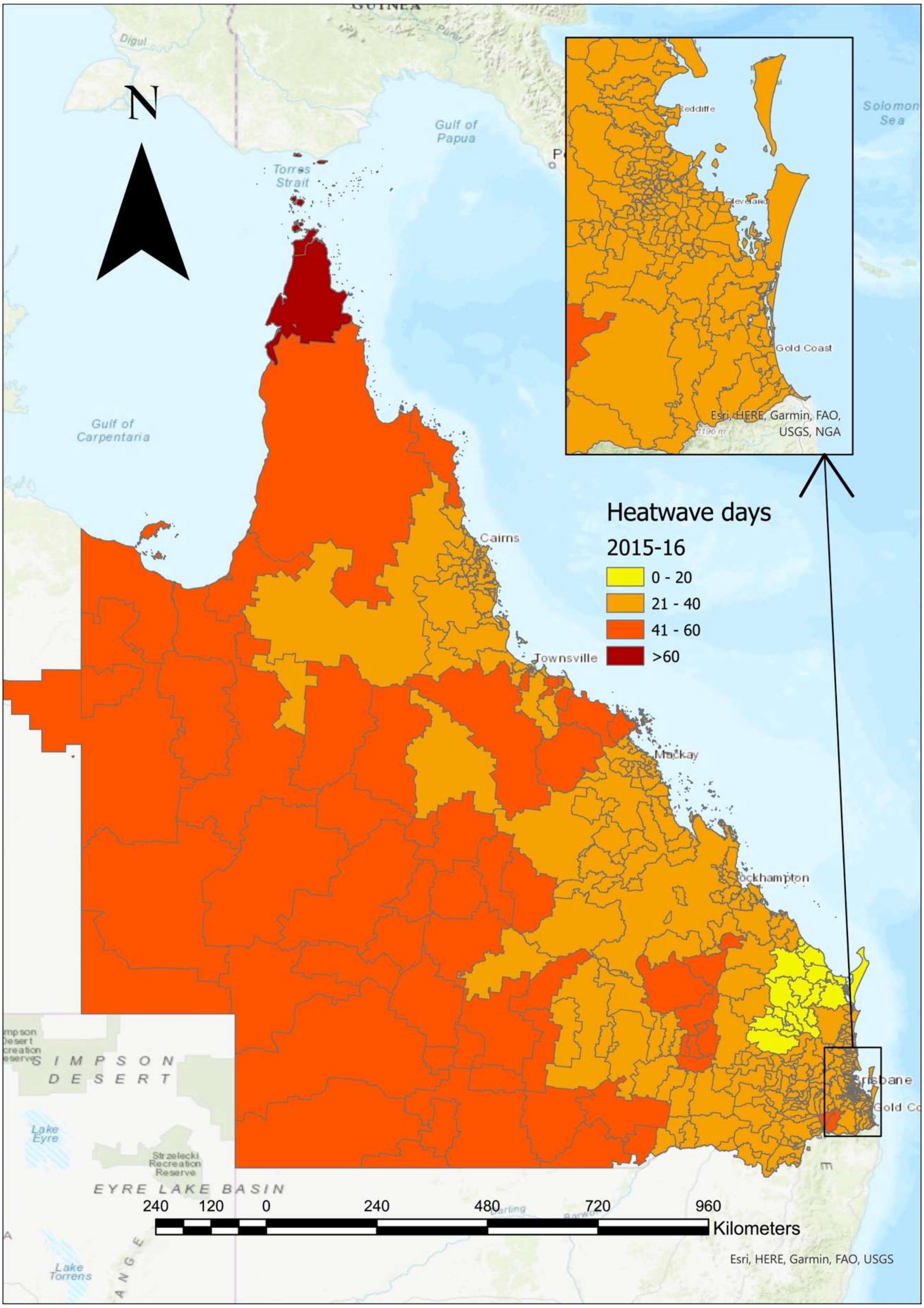

Figure S8- Number of heatwave days per postcode (Queensland; 2016-2017).

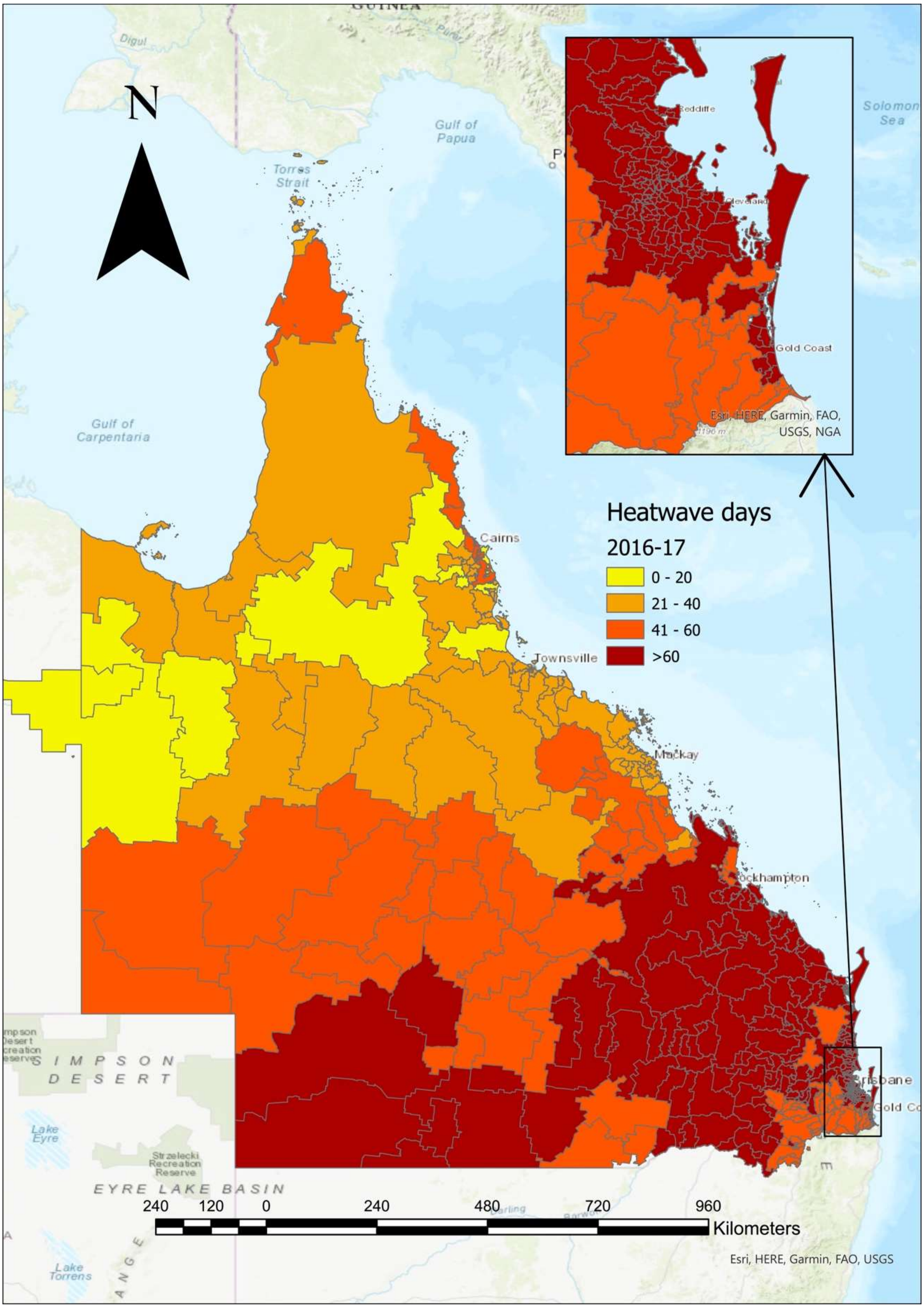

Figure S9- Number of heatwave days per postcode (Queensland; 2017-2018).

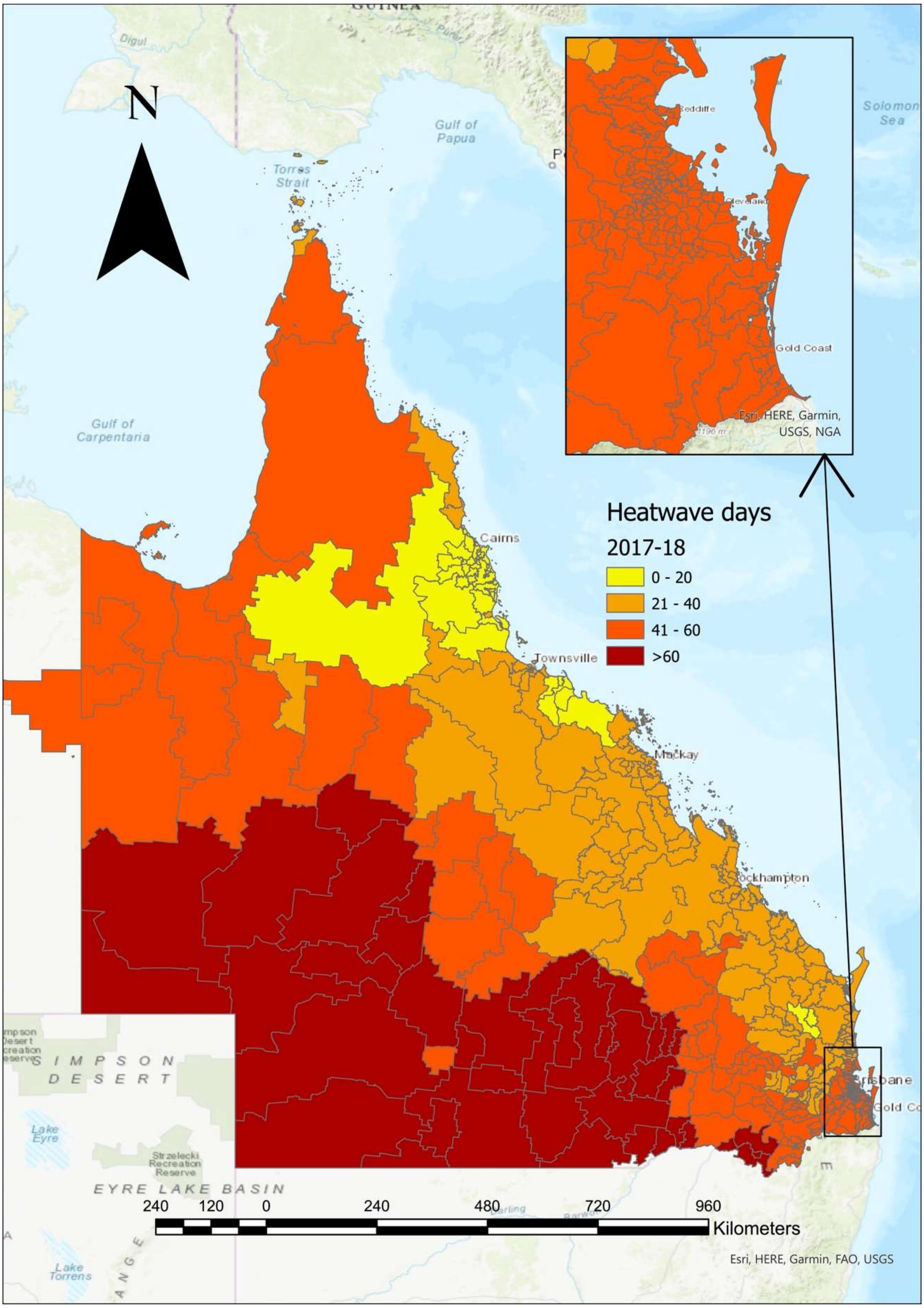

Figure S10- Number of heatwave days per postcode (Queensland; 2018-2019).

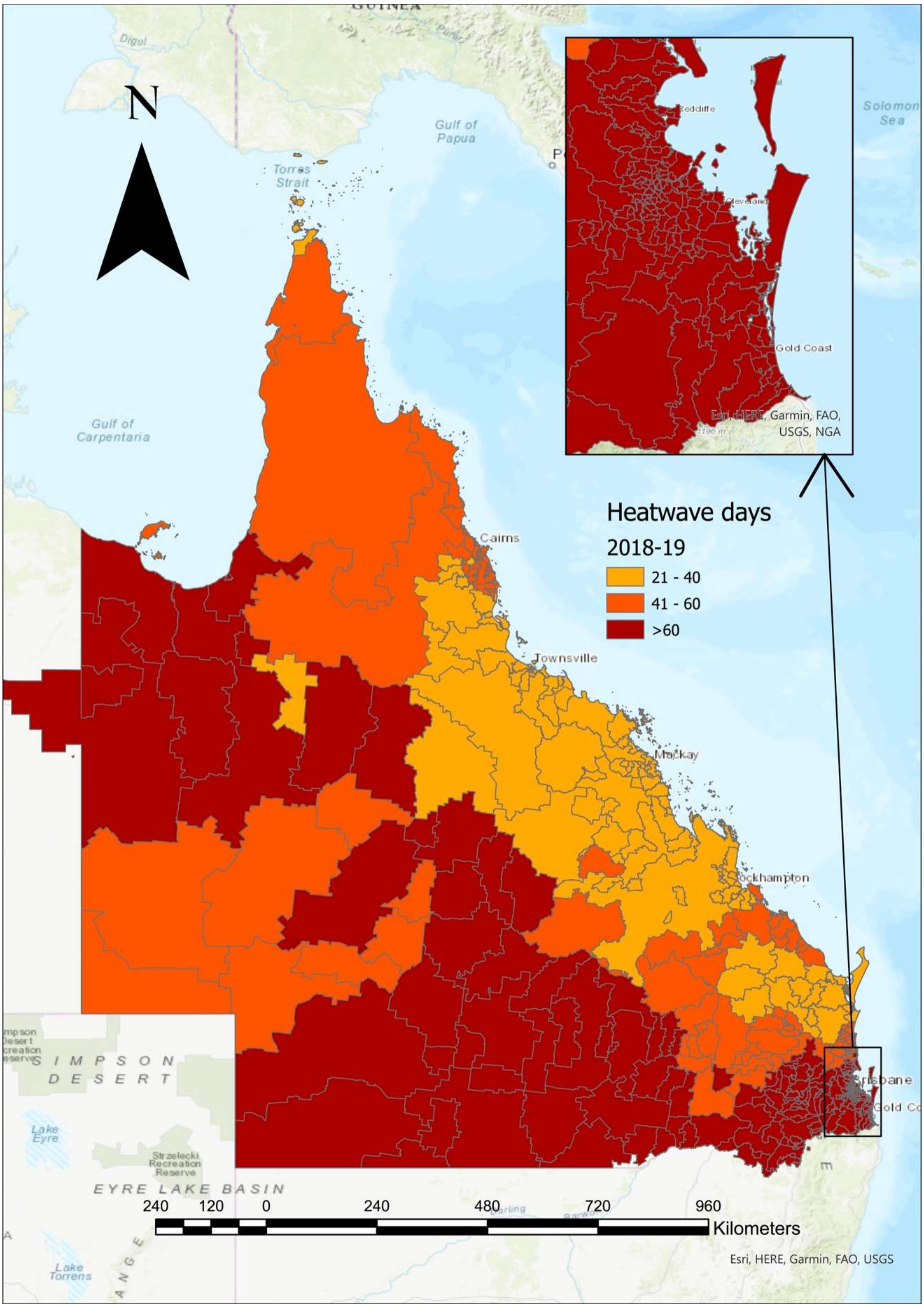

Figure S11- Proportion of ambulance calls by hour of the day for heatwaves vs. non-heatwave days (Queensland; 01 January 2010 to 31 December 2019)

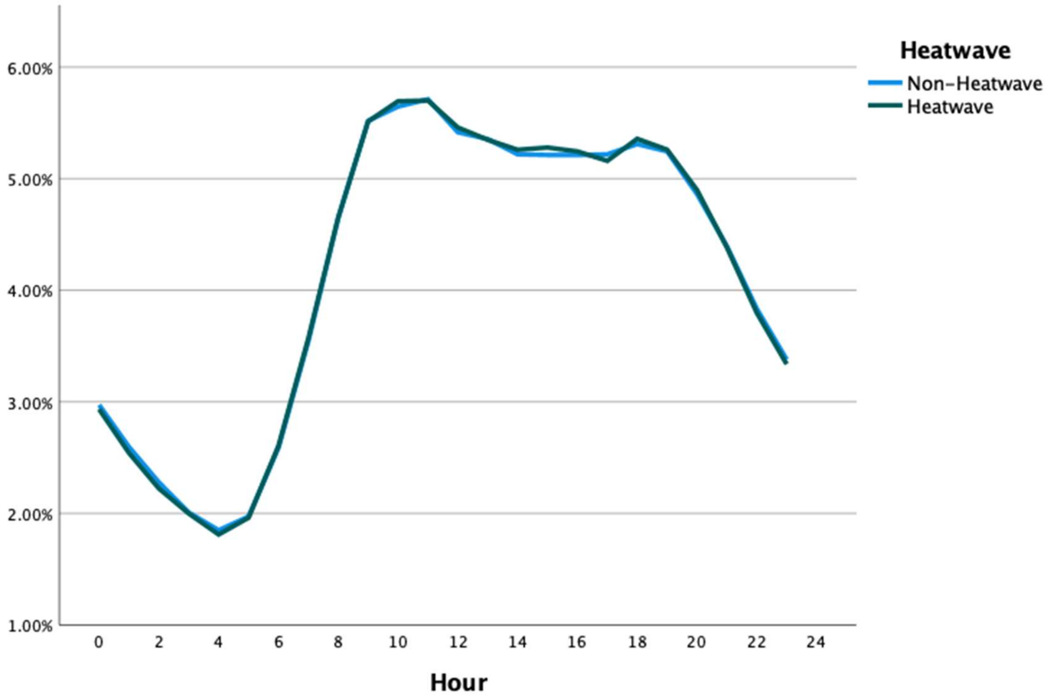

Figure S12- Crosstabulation and chi-square analysis of heatwave by hour of the day

Hour * Heatwave Crosstabulation

			Heatwave		
			0	1	Total
Hour	0	Count	52163	21724	73887
		% within Heatwave	3.0%	2.9%	3.0%
	1	Count	45546	18820	64366
		% within Heatwave	2.6%	2.5%	2.6%
	2	Count	39984	16462	56446
		% within Heatwave	2.3%	2.2%	2.3%
	3	Count	35244	14800	50044
		% within Heatwave	2.0%	2.0%	2.0%
	4	Count	32447	13409	45856
		% within Heatwave	1.8%	1.8%	1.8%
	5	Count	34612	14524	49136
		% within Heatwave	2.0%	2.0%	2.0%
	6	Count	45425	19292	64717
		% within Heatwave	2.6%	2.6%	2.6%
	7	Count	62199	26429	88628
		% within Heatwave	3.5%	3.6%	3.6%
	8	Count	81582	34460	116042
		% within Heatwave	4.6%	4.7%	4.7%
	9	Count	96850	40826	137676
		% within Heatwave	5.5%	5.5%	5.5%
	10	Count	99096	42163	141259
		% within Heatwave	5.6%	5.7%	5.7%
	11	Count	100240	42210	142450
		% within Heatwave	5.7%	5.7%	5.7%
	12	Count	95056	40422	135478
		% within Heatwave	5.4%	5.5%	5.4%
	13	Count	93985	39603	133588
		% within Heatwave	5.4%	5.3%	5.4%
	14	Count	91584	38942	130526
		% within Heatwave	5.2%	5.3%	5.2%
	15	Count	91488	39090	130578
		% within Heatwave	5.2%	5.3%	5.2%
	16	Count	91474	38833	130307
		% within Heatwave	5.2%	5.2%	5.2%
	17	Count	91579	38207	129786
		% within Heatwave	5.2%	5.2%	5.2%
	18	Count	93199	39670	132869
		% within Heatwave	5.3%	5.4%	5.3%
	19	Count	92050	38949	130999
		% within Heatwave	5.2%	5.3%	5.2%
	20	Count	85339	36274	121613
		% within Heatwave	4.9%	4.9%	4.9%
	21	Count	77132	32461	109593
		% within Heatwave	4.4%	4.4%	4.4%
	22	Count	67292	28119	95411
		% within Heatwave	3.8%	3.8%	3.8%
	23	Count	59298	24716	84014
		% within Heatwave	3.4%	3.3%	3.4%
Total		Count	1754864	740405	2495269
		% within Heatwave	100.0%	100.0%	100.0%

Chi-Square Tests

	Value	df	Asymptotic Significance (2-sided)
Pearson Chi-Square	44.357[a]	23	.005
Likelihood Ratio	44.405	23	.005
Linear-by-Linear Association	3.575	1	.059
N of Valid Cases	2495269		

a. 0 cells (0.0%) have expected count less than 5. The minimum expected count is 13606.55.

Figure S13- Crosstabulation and chi-square analysis of heatwave by day of the week

Heatwave * Monday=1, Sunday= 7 Crosstabulation

			Heatwave		
			0	1	Total
Monday=1, Sunday= 7	1.00	Count	254868_{a}	107017_{a}	361885
		Expected Count	254505.2	107379.8	361885.0
	2.00	Count	250518_{b}	103080_{b}	353598
		Expected Count	248677.2	104920.8	353598.0
	3.00	Count	253208_{c}	109972_{c}	363180
		Expected Count	255416.0	107764.0	363180.0
	4.00	Count	242264_{d}	108915_{d}	351179
		Expected Count	246975.9	104203.1	351179.0
	5.00	Count	253195_{e}	97188_{e}	350383
		Expected Count	246416.1	103966.9	350383.0
	6.00	Count	$248289_{a, b}$	$103368_{a, b}$	351657
		Expected Count	247312.1	104344.9	351657.0
	7.00	Count	252522_{c}	110865_{c}	363387
		Expected Count	255561.5	107825.5	363387.0
Total		Count	1754864	740405	2495269
		Expected Count	1754864.0	740405.0	2495269.0

Each subscript letter denotes a subset of Monday=1, Sunday= 7 categories whose column proportions do not differ significantly from each other at the .05 level.

Chi-Square Tests

	Value	df	Asymptotic Significance (2-sided)
Pearson Chi-Square	1178.279[a]	6	<.001
Likelihood Ratio	1183.242	6	<.001
Linear-by-Linear Association	4.139	1	.042
N of Valid Cases	2495269		

a. 0 cells (0.0%) have expected count less than 5. The minimum expected count is 103966.88.

Figure S14- Crosstabulation and chi-square analysis of heatwave by medical condition

BroadMPDS * Heatwave Crosstabulation

			Heatwave		
			0	1	Total
BroadMPDS	Cardiac	Count	206837$_a$	85673$_b$	292510
		Expected Count	205715.4	86794.6	292510.0
	Cold exposure	Count	84$_a$	37$_a$	121
		Expected Count	85.1	35.9	121.0
	Heat exposure	Count	2096$_a$	3278$_b$	5374
		Expected Count	3779.4	1594.6	5374.0
	Injuries	Count	481238$_a$	193272$_b$	674510
		Expected Count	474367.0	200143.0	674510.0
	Mental Health	Count	80745$_a$	37567$_b$	118312
		Expected Count	83206.0	35106.0	118312.0
	Obstetric	Count	16218$_a$	6586$_b$	22804
		Expected Count	16037.5	6766.5	22804.0
	Other/Transport	Count	247108$_a$	106931$_b$	354039
		Expected Count	248987.3	105051.7	354039.0
	Respiratory	Count	128722$_a$	51917$_b$	180639
		Expected Count	127039.2	53599.8	180639.0
	Specified Medical	Count	563287$_a$	243322$_b$	806609
		Expected Count	567269.1	239339.9	806609.0
	Stroke	Count	28529$_a$	11822$_a$	40351
		Expected Count	28377.9	11973.1	40351.0
Total		Count	1754864	740405	2495269
		Expected Count	1754864.0	740405.0	2495269.0

Each subscript letter denotes a subset of Heatwave categories whose column proportions do not differ significantly from each other at the .05 level.

Chi-Square Tests

	Value	df	Asymptotic Significance (2-sided)
Pearson Chi-Square	3355.065[a]	9	.000
Likelihood Ratio	3080.295	9	.000
N of Valid Cases	2495269		

a. 0 cells (0.0%) have expected count less than 5. The minimum expected count is 35.90.
